# [^18^F]DOPA PET/ceCT in diagnosis and staging of primary medullary thyroid carcinoma prior to surgery

**DOI:** 10.1007/s00259-018-4045-9

**Published:** 2018-05-15

**Authors:** Sazan Rasul, Sabrina Hartenbach, Katharina Rebhan, Adelina Göllner, Georgios Karanikas, Marius Mayerhoefer, Peter Mazal, Marcus Hacker, Markus Hartenbach

**Affiliations:** 1 Department of Biomedical Imaging and Image-Guided Therapy, Division of Nuclear Medicine, Medical University of Vienna, Waehringer-Guertel 18-20, A-1090 Vienna, Austria; 2 HistoConsultingHartenbach, Ulm, Germany; 3 Department of Biomedical Imaging and Image-Guided Therapy, Division of General and Pediatric Radiology, Medical University of Vienna, Vienna, Austria; 4 Clinical Institute of Pathology, Medical University of Vienna, Vienna, Austria

**Keywords:** Primary medullary thyroid carcinoma, [^18^F]DOPA PET/CT, Thyroid cancer, Calcitonin, Tumour staging

## Abstract

**Purpose:**

Medullary thyroid carcinoma (MTC) is characterized by a high rate of metastasis. In this study we evaluated the ability of [^18^F]DOPA PET/ceCT to stage MTC in patients with suspicious thyroid nodules and pathologically elevated serum calcitonin (Ctn) levels prior to total thyroidectomy and lymph node (LN) dissection.

**Methods:**

A group of 32 patients with sonographically suspicious thyroid nodules and pathologically elevated basal Ctn (bCtn) and stimulated Ctn (sCtn) levels underwent DOPA PET/ceCT prior to surgery. Postoperative histology served as the standard of reference for ultrasonography and DOPA PET/ceCT region-based LN staging. Univariate and multivariate regression analyses as well as receiver operating characteristic analysis were used to evaluate the correlations between preoperative and histological parameters and postoperative tumour persistence or relapse.

**Results:**

Primary MTC was histologically verified in all patients. Of the 32 patients, 28 showed increased DOPA decarboxylase activity in the primary tumour (sensitivity 88%, mean SUVmax 10.5). Undetected tumours were exclusively staged pT1a. The sensitivities of DOPA PET in the detection of central and lateral metastatic neck LN were 53% and 73%, in contrast to 20% and 39%, respectively, for neck ultrasonography. Preoperative bCtn and carcinoembryonic antigen levels as well as cN1b status and the number of involved neck regions on DOPA PET/ceCT were predictive of postoperative tumour persistence/relapse in the univariate regression analysis (*P* < 0.05). Only DOPA PET/ceCT cN1b status remained significant in the multivariate analysis (*P* = 0.016, relative risk 4.02).

**Conclusion:**

This study revealed that DOPA PET/ceCT has high sensitivity in the detection of primary MTC and superior sensitivity in the detection of LN metastases compared to ultrasonography. DOPA PET/ceCT identification of N1b status predicts postoperative tumour persistence. Thus, implementation of a DOPA-guided LN dissection might improve surgical success.

## Introduction

Medullary thyroid cancer (MTC) is a rare type of neuroendocrine tumour, that accounts for less than 5% of all thyroid cancer cases. It originates from C cells and is situated within the outer follicle walls of the thyroid gland. It occurs as either a familial or a sporadic form. The familial or hereditary form accounts for about 25% of all MTC and is inherited as an autosomal dominant trait due to the mutation and overexpression of the RET (REarranged during Transfection) proto-oncogene, which is located on chromosome 10 and causes mutations in the receptor tyrosine kinase. The sporadic form accounts for about 75% of all MTC and it mostly occurs in adults aged between 40 and 60 years [1]. Although MTC is commonly expected in patients with elevated basal (bCtn) and stimulated (sCtn) serum calcitonin levels, the final diagnosis is based on the results of preoperative fine-needle aspiration cytology and/or postoperative histological tissue analysis of a thyroid nodule or lymph node (LN) [2]. Surgical therapy by means of total thyroidectomy with bilateral neck dissection is still considered the gold standard curative therapy for MTC in all patients without distant metastases and without extranodal involvement [2]. Both bCtn and sCtn levels serve as tumour markers for postoperative follow-up of MTC patients and their elevation is an indicator of tumour recurrence and/or persistence [2], and estimation of Ctn doubling times (DT) can predict further tumour progression [3].

In addition, MTC is characterized by a high rate of metastasis. At the time of diagnosis, at least 50% of patients exhibit metastases to the LN with a 10-year survival of about 20–70% [4]. Therefore, precise preoperative diagnosis of MTC with exact localization of all involved LN is essential to obtain a curative surgical result with the lowest rate of recurrence postoperatively. Additionally, this will increase the survival rate in patients and help achieve optimal postoperative patient management, as has been shown in a prospective study by Modigliani et al. [5] that included 899 male and female MTC patients.

Moreover, the recent British Thyroid Association guidelines for the management of thyroid cancer recommend the use of neck ultrasonography, contrast-enhanced computed tomography (ceCT), magnetic resonance imaging (MRI) and bone scintigraphy for the systemic staging of MTC prior to surgery [2]. Neck ultrasonography is a very sensitive imaging method for identifying and characterizing thyroid lesions and can differentiate malignant from benign thyroid nodules in patients with suspected MTC [6]. Furthermore, previous studies have indicated the essential function of contrast-enhanced imaging approaches for differentiating malignant from benign solitary thyroid nodules [7]. On the other hand, ceCT enables identification and definitive localization of thyroid lesions in patients with MTC [8]. Concerning the challenging diagnostic issue of initial LN staging, metabolic changes in often morphologically very small metastatic MTC LNs might overcome the limitations of morphological imaging. This is supported by a small study by Golubic et al. [9], which found confirmed [^18^F]fluoro-l-dihydroxyphenylalanine (DOPA) PET/CT-positive MTC LN metastases in patients with persistently increased Ctn levels and negative on conventional imaging. Indeed, previous studies have demonstrated the crucial role of [^18^F]fluorodeoxyglucose (FDG) PET/CT in the early diagnosis of local and distant tumour recurrence during follow-up of patients with MTC [10–12]. Studies have also shown the value of DOPA PET/CT in the detection of recurrent or metastatic MTC after total thyroidectomy [13, 14]. In a prospective study in 26 patients, Beheshti et al. [14] found that DOPA PET/CT performs better than FDG PET/CT in the preoperative and follow-up assessment of MTC patients. This has also been shown recently in a study by Romero-Lluch et al. [15] which included 18 patients with recurrent MTC.

However, the majority of these previous studies using DOPA PET/CT were performed in patients with persistent or recurrent MTC postoperatively. There are only a few limited studies that have assessed the feasibility of DOPA PET/CT for identifying primary MTC and its related metastases prior to the first surgery [14]. Therefore, the aims of this study were to evaluate retrospectively the ability of DOPA PET/ceCT to detect primary MTC in patients with suspicious thyroid nodules and pathologically elevated serum Ctn levels and to compare the diagnostic reliability of DOPA PET/ceCT and neck ultrasonography for the initial staging of primary MTC prior to total thyroidectomy and LN dissection. A further aim of the study was to determine whether preoperative parameters such as age, sex, preoperative bCtn, sCtn and carcinoembryonic antigen (CEA) levels, cN status and number of involved neck regions on DOPA PET/CT, and the postoperative ratio of resected to metastatic LNs on histology are predictive of postoperative tumour persistence or recurrence.

## Materials and methods

### Patients

This retrospective single-centre study was approved by the Ethics Committee of the Medical University of Vienna (permit 1005/2018). All participants were recruited between 2008 and 2015 and gave written informed consent prior to the preoperative DOPA PET/CT examination. Thirty-two patients (50% women, aged 59 ± 13 years) with pathologically elevated bCtn (>10 pg/ml) and sCtn (>100 pg/ml) levels and sonographically suspicious thyroid nodules (31/32 patients) but no history of a previous thyroid surgery, were eligible for analysis.

### Laboratory parameters

Levels of bCtn, sCtn and CEA were measured in every patient preoperatively as well as postoperatively and during follow-up using commercially available assays. For determination of sCtn levels, the pentagastrin-stimulated Ctn test (Peptavlon®) was performed preoperatively in every patient with a pathologically elevated serum Ctn value of >10 pg/ml, and was repeated early (1–2 months) and then 12 months after surgery. Patients with persistently elevated Ctn levels 2 years after surgery underwent regular pentagastrin-stimulated Ctn testing. Patients with normal pentagastrin-stimulated Ctn test results 1 year after surgery were followed up yearly with bCtn and other disease-related laboratory measurements without further pentagastrin-stimulated Ctn testing. The median duration of follow-up was 5 years (range 2–8 years).

In all patients, the pentagastrin-stimulated Ctn tests were performed after fasting for about 12 h using a slow 10-s intravenous injection of 5 μg/kg body weight pentagastrin via a peripheral venous fixed cannula. Blood samples were obtained before and then 2, 3 and 10 min after the stimulation in every patient. Additionally, Ctn DT was determined using the established formula [DT × log/2 (log*A* − log*B*)] described by Miyauchi et al. [16]. According to the result, Ctn DT was divided into: 2–12 months, 12–24 months, and more than 24 months.

### Neck ultrasonography

In addition, thorough neck ultrasonography was performed preoperatively in each patient using a Toshiba Nemio XG ultrasound system. Nodules classified as sonographically suspicious were typically solid, hypoechogenic nodules without a halo but with microcalcifications, an irregular margin and increased blood perfusion. No fine-needle aspiration and/or evaluation of Ctn levels in the fine-needle aspiration washout fluid were performed in any of the patients prior to surgery due to the initially high bCtn and sCtn levels and suspicious thyroid nodules. According to the result of ultrasonography, LN metastases were classified as N0 (no detection of pathologically enlarged LN), N1a (detection of locoregional enlarged central neck LN, i.e. pretracheal, paratracheal and prelaryngeal; level VI), or N1b (detection of enlarged LN in the noncentral neck unilaterally or bilaterally; levels I to V).

### DOPA PET/CT examination

A preoperative [^18^F]DOPA PET/CT examination was also performed in the patients from the skull to the upper thighs using a 64-row multidetector hybrid system (Biograph TruePoint 64; Siemens, Erlangen, Germany) with an axial field-of-view of 216 mm, a PET sensitivity of 7.6 cps/kBq, and a transaxial PET resolution of 4–5 mm (full-width at half-maximum, FWHM). After surgery and during follow-up, DOPA PET/CT examinations with the same protocols as used before surgery were performed in patients with persistently elevated levels of bCtn and/or sCtn. All PET examinations were performed 60 min after intravenous administration of 3 MBq/kg body weight DOPA with 4 min/bed position, four iterations per 21 subsets, a 5-mm slice thickness and a 168 × 168 matrix, using the TrueX algorithm based on the point-spread function.

Venous-phase ceCT was performed after intravenous injection of 100 ml of a tri-iodinated, non-ionic contrast medium at a rate of 2 ml/s with a tube voltage of 120 mA, a tube current of 230 kV, a collimation of 64 × 0.6 mm, a 3-mm slice thickness at a 2-mm increment, and a 512 × 512 matrix. No premedication with carbidopa was administered in any patient prior to the DOPA PET/CT examination. Patients with bCT >500 pg/ml (*n* = 14) received an additional liver MRI examination [17].

The DOPA PET/CT examinations were evaluated by two experts in this field (a radiologist and a nuclear medicine specialist) who were aware of each patient’s Ctn levels. Local tumour diameter was measured as a metabolic axial approximation on PET/CT images supported by the use of CT soft-tissue alterations of the respective DOPA-positive thyroid nodules if applicable. DOPA-positive thyroid nodules and metastasis were defined as lesions that had visually higher [^18^F]DOPA uptake than the surrounding background activity. Accordingly, LN metastases were classified as N0 (no DOPA-positive LN), N1a (DOPA-positive LN around the thyroid, i.e. pretracheal, paratracheal, and prelaryngeal; all level VI), and N1b (DOPA-positive LN in the lateral neck; levels I to V).

### Surgery and histology

All patients underwent total thyroidectomy with modified neck dissection due to pathologically elevated bCtn and sCtn levels and the presence of suspicious thyroid nodules on ultrasonography (31/32 patients). The postoperative histological tissue examination of the removed thyroid gland and LN was performed according to pathohistological standards. The histological staging of the MTC followed the International Union Against Cancer (UICC) 2010 TNM Classification of Malignant Tumours (7th edition). Therefore, the histological results in patients recruited before 2010 were reclassified concerning T1a/b status to be comparable with the histological results in patients recruited after 2010.

Postoperatively, persistence of MTC was defined as persistently elevated serum bCtn and sCtn levels with evidence of local and/or distant metastases on postoperative DOPA PET/CT, whereas recurrence of MTC was defined as re-elevation of serum bCtn and sCtn levels with the presence of local and/or distant metastases on postoperative DOPA PET/CT after a period of improvement of the disease.

### Statistical analysis

The collected clinical characteristics of the studied patients were evaluated using SPSS, version 24.0 (IBM Corp., Armonk, NY). Data were log10-transformed for all statistical analyses, and were analysed with the Kolmogorov–Smirnov test to identify their distribution. Non-normally distributed data are expressed as medians and ranges. Univariate and multivariate Cox regression analysis was adjusted for age, sex, preoperative bCtn, sCtn and CEA levels. cN status and number of involved neck regions on DOPA PET/CT, and the postoperative ratio of resected to metastatic LNs on histology. Two-way contingency table analyses were also performed to define the sensitivity, specificity, accuracy, positive predictive value (PPV) and negative predictive value (NPV) of preoperative DOPA PET/CT and neck ultrasonography in relation to the results of postoperative histology. For all statistical tests, values of *P* <0.05 were considered statistically significant.

## Results

Median (interquartile range) preoperative bCtn, sCtn and CEA values were 208 pg/ml (87–1,143 pg/ml), 2,950 pg/ml (1,798–27,356 pg/ml) and 19.4 μg/ml (5.2–58.2 μg/ml). Clinical characteristics as well as levels of bCtn, sCtn and CEA in each patient preoperatively, postoperatively and during follow-up are presented in Table 1.

**Table 1:** Clinical characteristic of the studied patients

Patient number	Gender	Age (years)	Before surgery			After surgery						Calcitonin doubling time (months)	Disease prognosis
						1–2 months			12 months				
			bCtn (pg/ml)	sCtn (pg/ml)	CEA (μg/ml)	bCtn (pg/ml)	sCtn (pg/ml)	CEA (μg/ml)	bCtn (pg/ml)	sCtn (pg/ml)	CEA (μg/ml)		
1	M	67	1,363	25,145	24.5	0	0	1.7	0	0	1.5	–	–
2	F	79	721	2,950	18.6	0	0	3.4	0	0	3.2	–	–
3	F	69	22.5	434	2.5	0	0	1.8	0	1	1.8	–	–
4	M	53	1,176	52,683	54.8	145	3,300	9.7	129	1,619	7.9	NC	P
5	M	67	41	1,330	433.4	5,658	244,000	44.2	15,291	244,000	358.7	2−12	P
6	M	68	116	2,744	29.3	0	0	1.1	0	0	1.8	–	–
7	F	65	12,411	59,059	193.5	351	1,338	31.2	701	3,668	265.2	2−12	P
8	M	61	1,942	29,568	80.1	466	4,889	25.5	498	4,889	24.9	>24	P
9	M	56	211	2,198	3.7	0	0	1.3	0	0	1.2	–	–
10	M	72	1,110	12,043	18.9	68.2	779	4.1	154	1,640	3.3	2−12	P
11	M	71	202	12,373	21.2	0	0	2.4	0	0	1.6	–	–
12	F	51	863	61,036	70.7	0	0	35	0	0	3.5	–	–
13	M	56	16.8	979	2.5	0	0	2.2	0	0	1.9	–	–
14	F	41	676	4,746	38.5	0	0	25.7	0	0	1.0	–	–
15	F	70	110	1,060	5	0	0	1.2	0	0	1.1	–	–
16	M	30	15,477	209,186	413.3	935	9,179	39.6	1,272.0	17,393	48.3	2−12	P
17	F	78	84	1,902	9.9	0	0	0.8	219	341	23.9	2−12	R
18	F	52	144	2,491	3.7	0	0	1.2	0	0	1.1	–	–
19	F	76	431	1,871	7.2	78	114	1.7	86	184.6	1.5	>24	P
20	F	29	204.8	10,262	20.3	0.9	39.2	4.2	1.3	22	0.7	>24	P
21	F	47	1,862	8,917	68.2	591	1,281	32.7	672	2,425	44.7	>24	P
22	F	69	16.7	572	10.1	0	0	3.34	0	0	2.6	–	–
23	F	41	1,670	55,177	70.7	1,326	26,636	51.7	608.0	30,858	56.2	NC	P
24	M	60	86.1	602	19.7	0	0	5.48	0	0	2.2	–	–
25	M	55	894	75,366	49.7	0	0	1.9	0	0	1.8	–	–
26	M	45	417.7	1,827	19.1	19.2	228.3	2.3	28	360	2.6	12−24	P
27	F	50	96.6	827.8	5.3	0	0	2.7	0	0	2.1	–	–
28	M	61	46	1,990	1.2	0	0	1.4	0	0	1.0	–	–
29	F	51	44.1	439	4.5	0	0	1.7	0	0	1.5	–	–
30	M	48	87.7	1,769	1.7	13.8	398	0	8.1	365	0	2−12	P
31	M	76	17,330	56,530	878.4	23,647	NA	1426	63,106.0	NA	5,229	2−12	P
32	F	63	93.5	3,563	9.8	4	4.7	2.6	2.7	3.6	2.6	–	–^a^

*M* male, *F* female, *bCtn* basal calcitonin, *sCtn* stimulated calcitonin, *CEA* carcinoembryonic antigen, *P* persistence, *R* recurrence, *NA* not available, *NC* not calculable

^a^Patient with measurable calcitonin but no evidence of pathology on postoperative DOPA PET/CT or during follow-up

### Surgery and postoperative histology

Surgically, total thyroidectomy with central and bilateral neck dissection was performed in 23 patients (72%) as standard at our institution in this cohort of patients with high Ctn levels. In 9 patients (28%), however, total thyroidectomy with only central neck dissection was carried out due to negative imaging results, lower bCtn levels, and individual decisions. Moreover, postoperative histological tissue analysis revealed the diagnosis of MTC in all 32 studied patients. The histological TNM classifications of the removed thyroid glands were as follows: T1a in 12 patients, T1b in 9 patients, T2 in 4 patients, and T3b in 7 patients. The histological TNM classifications of the removed neck LNs were as follows: N0 status in 13 patients, N1a in 4 patients, and N1b in 15 patients (Table 2). Furthermore, in 8 of the 15 patients with stage N1b, histological results indicated bilateral involvement of cervical LN. Overall, 2,547 LNs (83 ± 46 per patient, mean ± SD) were resected from all studied patients, and of these, 317 showed malignancy.

**Table 2:** Sensitivity of neck ultrasonography and [^18^F]DOPA in the detection of regional and locoregional lymph node metastasis in all studied patients in relation to the results of histology

	DOPA PET/CT	Neck ultrasonography	Histology
Central regions (level VI)			
Metastatic levels (*n*)	8	3	15
Sensitivity	53% (95% CI 35–53)*	20% (95% CI 7–20)	
Specificity	100% (95% CI 84–100)	100% (95% CI 88–100)	
PPV	100% (95% CI 65–100)	100% (95% CI 33–100)	
NPV	71% (95% CI 59–71)*	59% (95% CI 52–59)	
Accuracy	78% (95% CI 61–78)	63% (95% CI 50–63)	
* P* value vs. histology	0.001*	0.092	
Lateral regions (levels I, II, III, IV, V)			
Metastatic levels (*n*)	19	10	26
Sensitivity	73% (95% CI 60–73)*	39% (95% CI 26–39)	
Specificity	100% (95% CI 99–100)	100% (95% CI 99–100)	
PPV	100% (95% CI 82–100)	100% (95% CI 68–100)	
NPV	97% (95% CI 96–97)*	94% (95% CI 93–94)	
Accuracy	98% (95% CI 95–98)*	94% (95% CI 92–94)	
* P* value vs. histology	<0.001	<0.001	
All regions			
Metastatic levels (*n*)	27	13	41
Sensitivity	66% (95% CI 57–66)*	32% (95% CI 23–32)	
Specificity	100% (95% CI 99–100)	100% (95% CI 99–100)	
PPV	100% (95% CI 86–100)	100% (95% CI 73–100)	
NPV	95% (95% CI 94–95)*	91% (95% CI 90–91)	
Accuracy	96% (95% CI 93–96)*	91% (95% CI 89–91)	
* P* value vs. histology	<0.001	<0.001	
Patient-based analysis			
Patients with LN metastases (*n*)	12	8	19
Sensitivity	63% (95% CI 48–63)*	42% (95% CI 27–42)	
Specificity	100% (95% CI 78–100)	100% (95% CI 78–100)	
PPV	100% (95% CI 76–100)	100% (95% CI 65–100)	
NPV	65% (95% CI 50–65)	54% (95% CI 43–54)	
Accuracy	78% (95% CI 60–78)	66% (95% CI 48–66)	
* P* value vs. histology	<0.001	0.01	
Patients with N0 (*n*)	20	24	13
Patients with N1a (*n*)	1	1	4
Patients with N1b (*n*)	11	7	15
Patients with N1a+b (*n*)	7	2	11
Patients with bilateral LN metastasis (*n*)	6	1	8

*LN* lymph node, *PPV* positive predictive value, *NPV* negative predictive value

*P < 0.05 vs. ultrasonography

### Preoperative neck ultrasonography

Among the 32 studied patients, 31 presented with at least one sonographically suspicious thyroid nodule in one or both thyroid lobes. However, there was no evidence of a definable thyroid nodule in only one patient. Moreover, 23 patients showed no evidence of pathologically enlarged or morphologically suspicious LN and were therefore staged N0. Two patients showed suspicious LN at neck level VI and were staged N1a, and six patients showed unilateral suspicious LN in the cervical region and were staged N1b (Table 2). In only one patient did neck ultrasonography show bilateral suspicious LN in the cervical region, and the patient was staged N1b. Patient-based and region-based analyses as well as central and lateral evaluations are shown in Table 2, demonstrating even nonsignificant results in the central neck region.

### Preoperative DOPA PET/CT

**Fig. 1:**
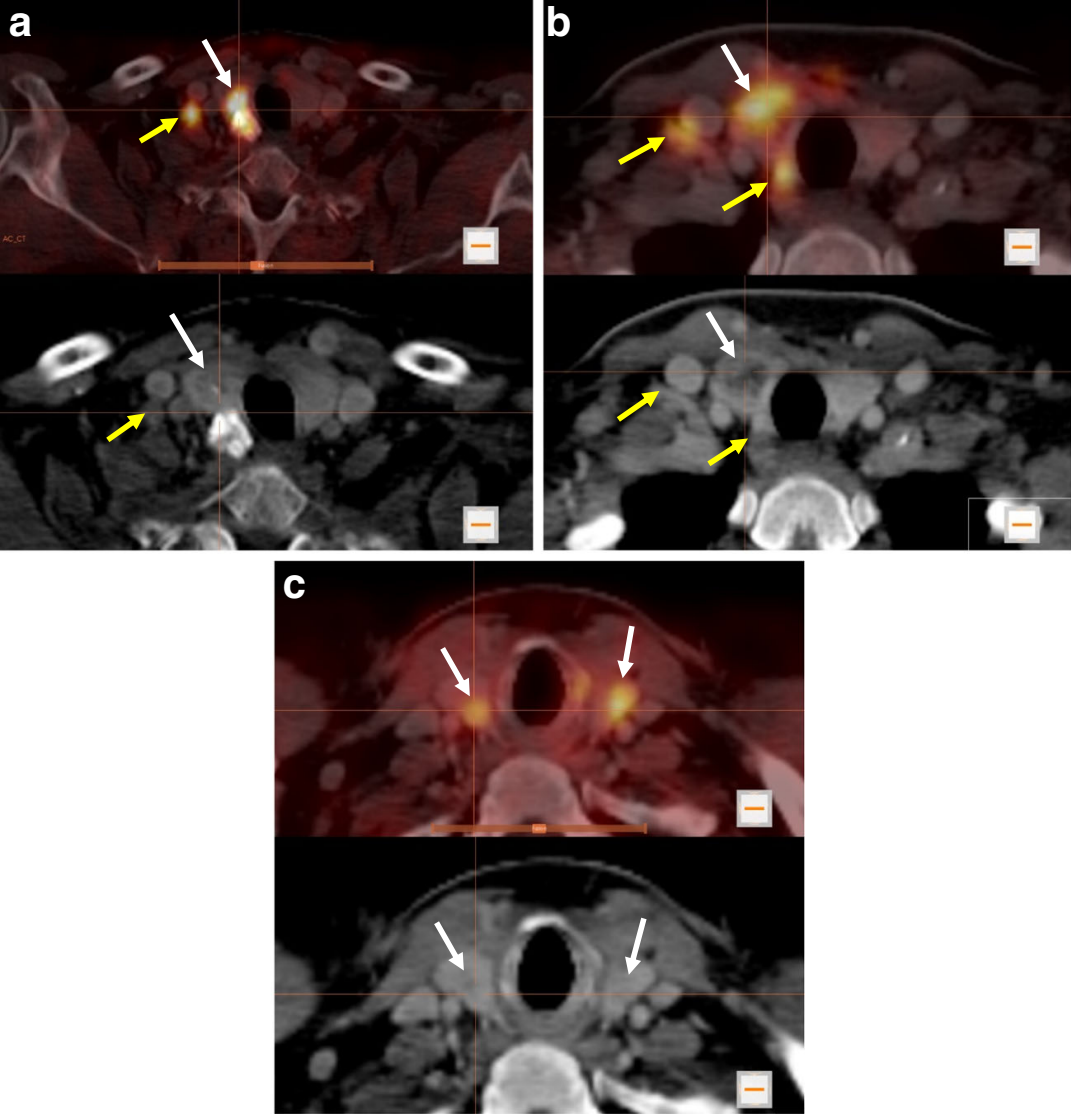
Initial [^18^F]DOPA PET/CT in patients with suspicious thyroid nodules prior to total thyroidectomy and lymph nodes dissection. **a** Increased DOPA uptake in the primary tumour in the right thyroid lobe (*white arrows*) and in a right lateral neck lymph node at level IV (*yellow arrows*) in a 61-year-old male patient. **b** Increased DOPA uptake in the primary tumour in the right thyroid lobe (*white arrows*), in a lateral neck lymph node at level IV and in a central (paratracheal) lymph node at level VI (*yellow arrows*) in a 47-year-old female patient. **c** Increased DOPA uptake in the primary tumour in both thyroid lobes (*white arrows*) in a 48-year-old male patient

**Fig. 2:**
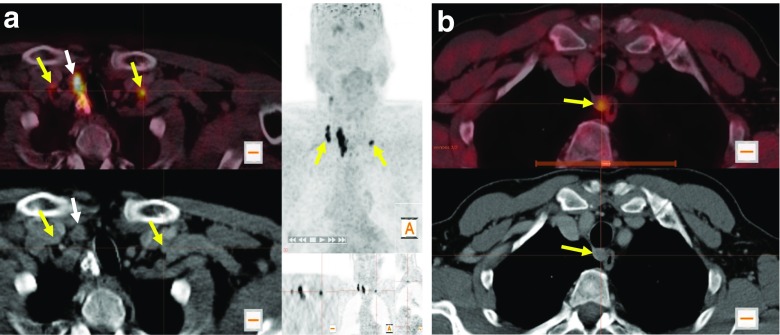
Initial [^18^F]DOPA PET/CT examinations in (**a**) a 61-year-old male patient with increased DOPA uptake in right and left level IV LNs (*yellow arrows*), while neck ultrasonography showed suspicious lymph nodes only on the right side, and (**b**) a 72-year-old male patient with increased DOPA uptake in a lymph node at lower level VI that was not been seen with neck ultrasonography

**Fig. 3:**
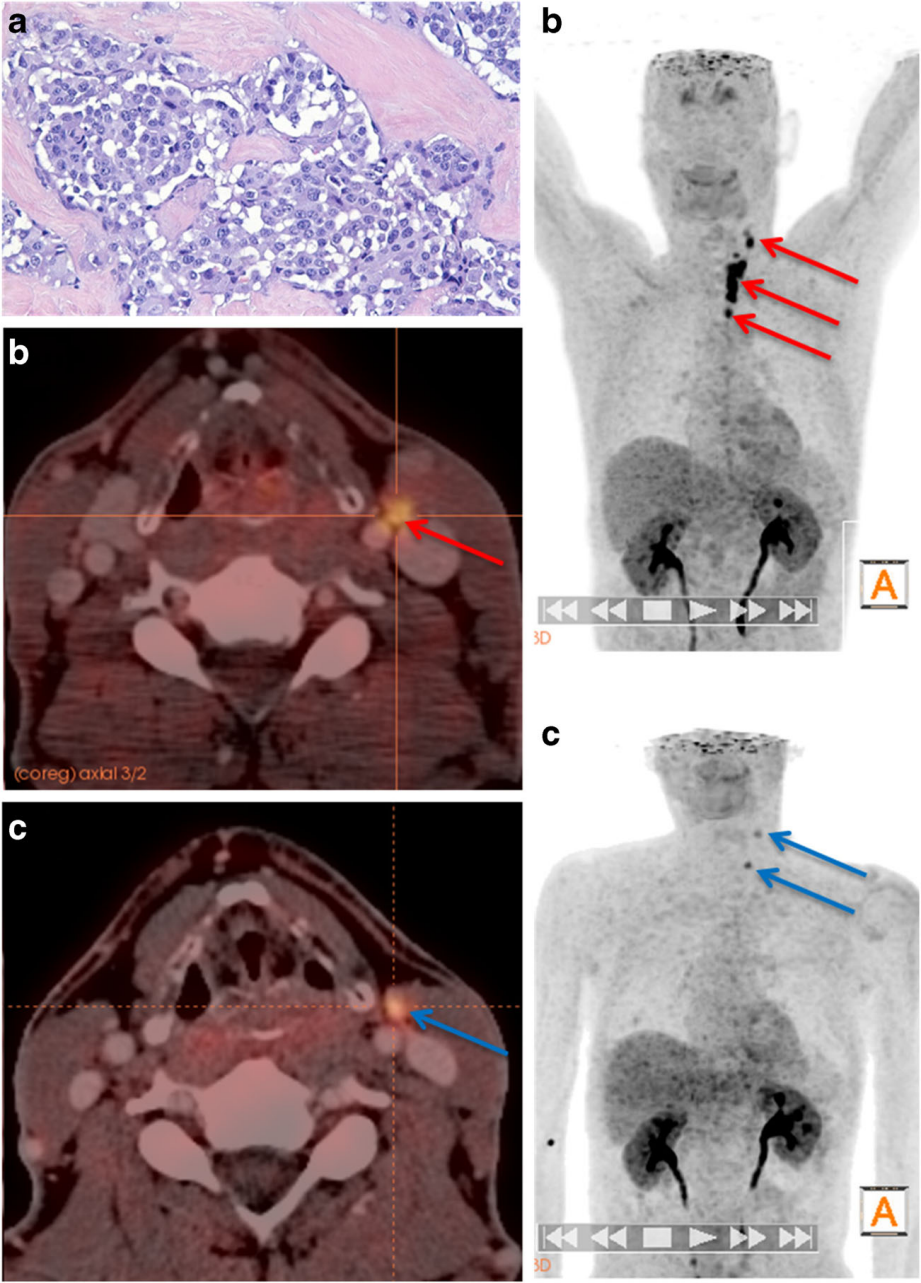
A 53-year-old male patient with a histologically proven medullary thyroid carcinoma (MTC) after thyroidectomy. **a** Histology shows MTC with desmoplastic stroma reaction (H&E, ×400). **b** Preoperative [^18^F]DOPA PET/CT shows focally increased [^18^F]DOPA uptake in the left thyroid lobe and in multiple lymph nodes of the central and lateral neck (N1a+b). **c** [^18^F]DOPA PET/CT in the same patient after total thyroidectomy and lymph node dissection with persistently elevated basal calcitonin levels still shows increased [^18^F]DOPA uptake in residual level IIa and level IV lymph nodes on the left

[^18^F]DOPA PET/CT showed increased DOPA decarboxylase activity in the primary tumour of 28 patients with a maximum SUV (SUVmax.) of 10.5 ± 4.2 (mean ± standard deviation; representative examples are shown in Fig. 1a, b). One patient presented with bilateral suspicious thyroid nodules. Both nodules revealed increased DOPA decarboxylase activity (Fig. 1c). However, there was no evidence of increased DOPA decarboxylase activity in the primary tumour in only four patients. Preoperative LN staging with [^18^F]DOPA PET/CT showed N0 in 20 patients, N1a in 1 patient, and N1b in 11 patients. In 6 of the patients with stage N1b, [^18^F]DOPA showed bilateral suspicious LN in neck regions and in one patient showed suspicious LN in the superior mediastinum outside the neck region (Figs. 2a and 3b).

### Postoperative statistical evaluations

After surgery and in relation to the results of the postoperative histological tissue analysis, [^18^F]DOPA detected 88% of the primary MTCs. Patients with no evidence of increased DOPA decarboxylase activity in the primary tumour were exclusively those with T1a tumour stage. Concerning the LN staging, [^18^F]DOPA detected overall 27 of 41 regional LN detected on histology. [^18^F]DOPA had a higher sensitivity in detecting lateral LN (19 of 26) than central LN (8 of 15; 73% and 53%, respectively; Table 2). The sensitivity of [^18^F]DOPA PET/CT was significantly higher than that of ultrasonography in regional and patient-based LN assessments (Table 2). In addition, in six of eight patients with histologically verified bilateral involvement of cervical LN, [^18^F]DOPA showed bilateral LN with increased DOPA decarboxylase activity (Fig. 2a).

On the other hand, in comparison with histology, neck ultrasonography was able to define and determine the suspicious primary thyroid tumour in 31 of the 32 patients. Nevertheless, regarding regional LN staging, neck ultrasonography detected overall 13 of 41 LN regions detected on histology with a sensitivity of 32%. Of these, 3 of 15 were level VI regions and only 10 out of 26 were lateral regions, resulting in a sensitivity of 20% and 39%, respectively (Table 2). Furthermore, neck ultrasonography showed bilateral cervical LN involvement in only one of eight patients.

**Fig. 4:**
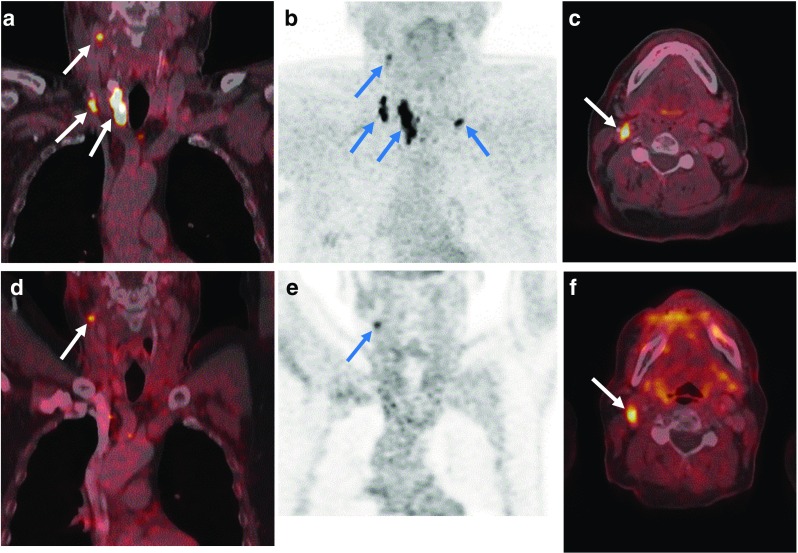
A 61-year-old male patient with persistently elevated basal calcitonin levels postoperatively. **a–c** Preoperative [^18^F]DOPA PET/CT images show increased DOPA uptake in the primary tumour in the right thyroid lobe as well as multiple bilateral DOPA-positive cervical lymph nodes (levels II, III, IV and VI). **d–f** Postoperative [^18^F]DOPA PET/CT images in the same patient 2 months after total thyroidectomy with central and bilateral neck dissection, show a residual lymph node with focal DOPA uptake in the right upper cervical region (level II)

**Fig. 5:**
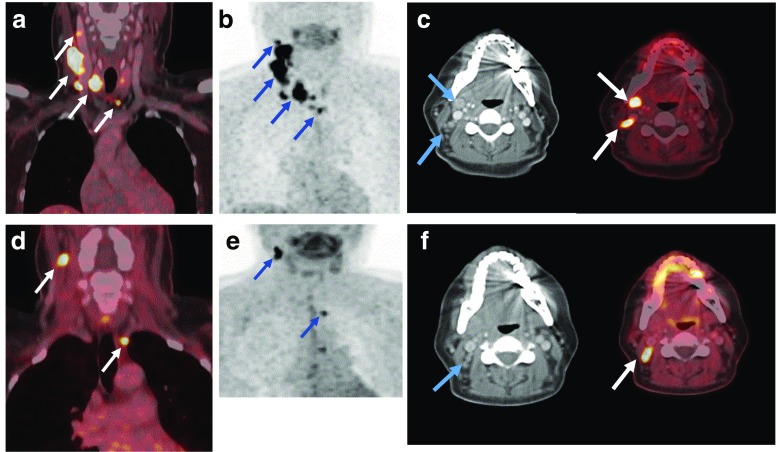
A 47-year-old female patient with further increasing basal calcitonin levels after surgery. **a–c** Preoperative [^18^F]DOPA PET/CT images show a DOPA-positive primary tumour in the right thyroid lobe with multiple DOPA-positive lymph nodes in the right cervical region (levels II, III, IV and V) as well as in the lower central region (level VI). **d–f** Postoperative [^18^F]DOPA PET/CT images in the same patient show persistently DOPA-positive lymph nodes in the right upper accessory level V and central level VI

**Fig. 6:**
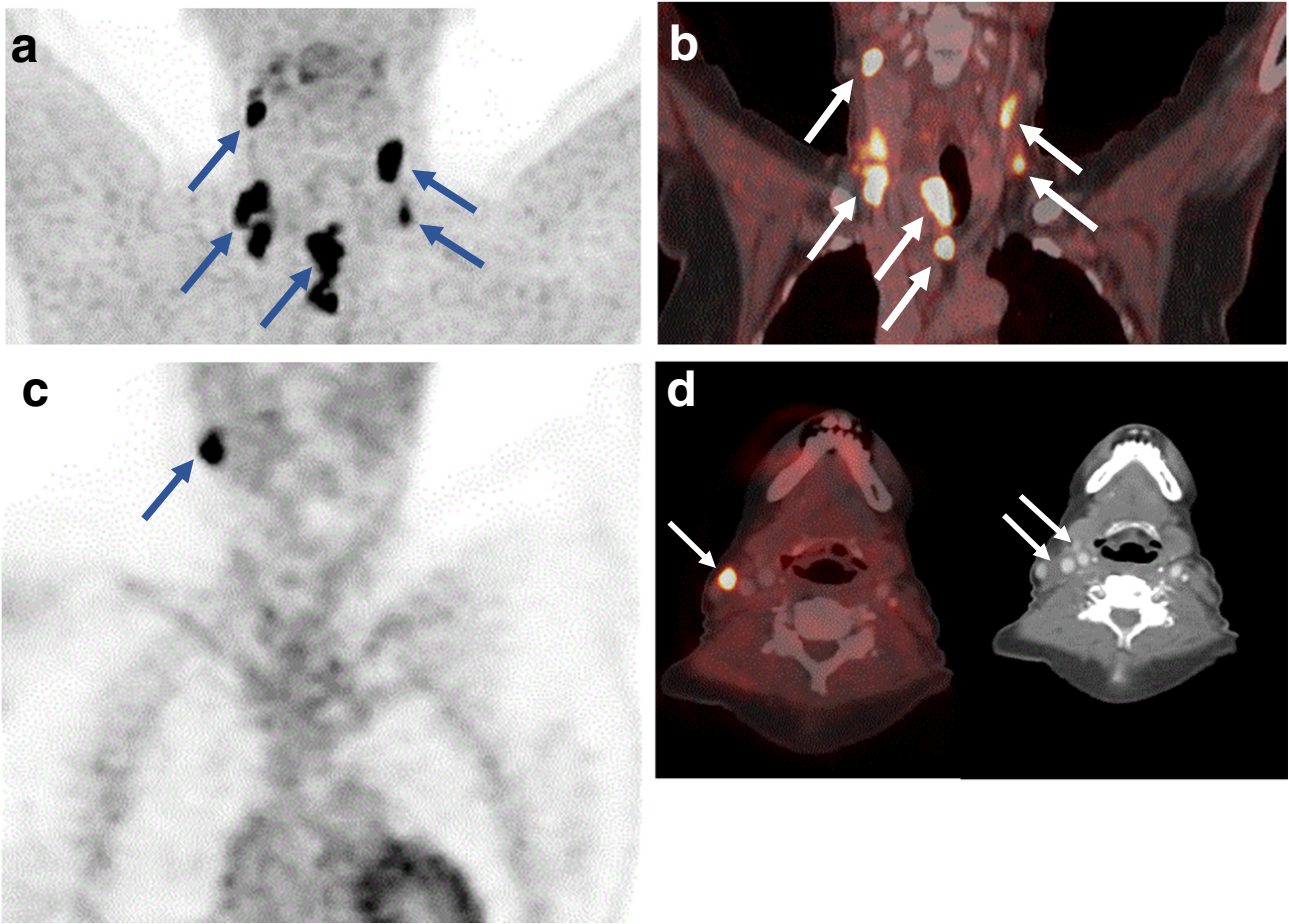
A 65-year-old female patient with MTC and bilateral lymph node metastases with persistently elevated levels of serum basal calcitonin after surgery. **a**, **b** Preoperative [^18^F]DOPA PET/CT images show DOPA-positive primary tumour in the right thyroid lobe with bilateral DOPA-positive lymph nodes in the cervical region (levels II, III and IV) and central region (level VI). **c**, **d** [^18^F]DOPA PET/CT images in the same patient 2 months after total thyroidectomy, and central and bilateral lymph node dissection, show a DOPA-positive residual lymph node in the upper right cervical region (level II)

Two months after surgery, 14 patients showed measurable bCtn levels and were categorized as having persistent MTC, while 17 patients showed no detectable levels of bCtn and sCtn. The median (range) postoperative bCtn, sCtn and CEA levels were 0.5 pg/ml (0–23,647 pg/ml), 0.5 pg/ml (0–244,000 pg/ml) and 2.6 μg/ml (0–1,426 μg/ml), respectively. LN with increased DOPA decarboxylase activity were detected on follow-up [^18^F]DOPA PET/CT examinations in these patients ([^18^F]DOPA PET/CT imaging in representative patients is shown in Figs. 3, 4, 5, and 6). The patient with the highest Ctn levels showed liver metastases on the first follow-up scan 2 months after surgery.

One year after surgery only one patient showed newly increased bCtn and sCtn levels; this patient was therefore categorized as having MTC relapse. Furthermore, among patients with persistent and re-elevated Ctn levels, seven showed a Ctn DT of 2–12 months, one a Ctn DT of 12–24 months and four a Ctn DT of more than 24 months (Table 1). During long-term follow-up of these patients, four patients with a Ctn DT of 2–12 months died within 2–6 years of initial diagnosis of MTC. However, the Ctn DT could not be calculatedlculated in two patients who underwent repeated neck surgery to remove metastatic LNs after the initial operation and showed gradually decreasing serum bCtn levels.

In the univariate Cox regression analysis, preoperative bCtn and CEA levels, cN1b status and the number of involved neck regions on [^18^F]DOPA PET/CT were predictors of postoperative tumour persistence, as was the ratio of resected to metastatic LNs on histology (*P* < 0.05). No significant associations were found for SUV parameters, LN status on ultrasonography or primary tumour status. In the multivariate regression analysis including the preoperative parameters significant in the univariate analysis (bCtn, CEA, [^18^F]DOPA PET/CT cN1b status and number of involved neck regions), only DOPA PET/CT cN1b status remained significant (*P* = 0.016, relative risk 4.02). This resulted in a sensitivity, specificity, accuracy, PPV and NPV of 79%, 100%, 91%, 100% and 81%, respectively (*P* < 0.001).

## Discussion

Thyroid cancer including MTC is one of the ten most common types of cancer worldwide, although MTC represents only a subgroup of about 3% of thyroid cancers [4]. Data on imaging in preoperative staging of primary MTC is almost unavailable and even Wells et al. in the section “Preoperative imaging studies” of the “Revised American Thyroid Association Guidelines for the Management of Medullary Thyroid Carcinoma” refer to imaging studies in recurrent or persistent MTCs [4]. We performed [^18^F]DOPA PET/CT examinations in patients with pathologically elevated bCtn and sCtn levels and suspected MTC preoperatively. Concerning the primary tumour, DOPA uptake was positive in 28 of the 32 patients (88%) with histologically verified MTC. In agreement with this result, smaller cohorts are available in the literature. Beheshti et al. [14] found a sensitivity of about 81% for DOPA PET/CT in the detection of the primary tumour in a cohort of eight patients with primary MTC. In a study by Hoegerle et al. [18], three primary/local recurrence MTC lesions showed DOPA uptake.

Regarding preoperative LN staging, although ultrasonography plays a critical role in the diagnosis of a primary thyroid tumour and is considered as an important and ubiquitously available imaging tool for detecting locoregional LN metastasis before and after total thyroidectomy [19], its role in the detection of nonlocoregional and distant LN metastasis is limited, and its performance is observer-dependent by the nature of the method itself. In our study, [^18^F]DOPA PET/CT showed significantly higher sensitivity (63%) in the identification of metastatic LN in the preoperative setting than neck ultrasonography (42%) in relation to the histological results. In an additional level-based evaluation, DOPA PET/CT performed significantly better than ultrasonography (sensitivities 66% and 32%, respectively). This was mainly driven by the sensitivity for lateral LN regions (73%) which was also significantly higher than with ultrasonography. In accordance with these findings, in a study in recurrent MTC patients, neck ultrasonography could not identify cervical LN metastases in more than 50% of patients and showed nonspecific and ambiguous results in the other patients, while [^18^F]DOPA PET revealed positive LN in 7 of 13 studied patients [15]. Similarly, Hoegerle et al. [18] and Treglia et al. [20] found that the sensitivity of [^18^F]DOPA PET in LN staging was higher than the sensitivities of [^18^F]FDG PET, somatostatin receptor scintigraphy and morphological tomographic imaging including CT and MRI.

However, with regard to LN staging, the [^18^F]DOPA PET results were false-negative in relation to the histological results in 7 of 19 patients, which represents 37% of the patients studied. In our region-based analysis, DOPA PET/CT was also only able to detect 53% of the centrally located LN metastases, which is significantly better than ultrasonography (20%) but still not an acceptable rate for a diagnostic method. In this respect, many factors such as LN size and localization as well as the time between intravenous injection of the radiopharmaceutical and the PET acquisition start time [14] could affect the efficacy and the tissue uptake of [^18^F]DOPA, and all these factors should be taken into consideration. Four patients had only one positive LN, two patients had two positive LNs each and one patient had five positive LNs. Nevertheless, four patients were classified pN1b, so that [^18^F]DOPA PET/CT was not able to rule out noncentral LN metastasis. However, [^18^F]DOPA PET/CT was able to stage 73% of the patients (11/15) correctly as N1b.

As surgery is the only method with curative intent in MTC [4], preoperative risk stratification might be almost as important as accurate staging. We found that the preoperative cN1b status on [^18^F]DOPA PET/CT, the number of involved LN regions and the preoperative levels of both bCtn and CEA are predictive of postoperative tumour persistence and recurrence. This has also been demonstrated in a larger cohort for bCtn with a comparable cut-off value of >1,000 pg/ml [21]. However, although our cohort was comparatively small for an outcome prediction analysis, DOPA PET/CT cN1b status was the only independent predictor of postoperative tumour persistence/recurrence in the multivariate analysis. The results suggest that [^18^F]DOPA PET/CT provides superior preoperative outcome prediction to the known tumour markers while additionally providing information about the respective LN locations. Nevertheless, in comparison to the postoperative [^18^F]DOPA PET/CT examinations (Figs. 3, 4, 5 and 6), postoperative persistence of MTC was mainly detected in cervical LNs already known from preoperative imaging. Reasonable explanations for this finding could be either that the small size of the involved LNs hampered the intraoperative detection process or that surgical nontargeted exploration of the involved LN level would have caused an unreasonable risk of sever side effects. Both issues might be addressed in the future using the higher detection rate of [^18^F]DOPA in a radioguided surgical approach which might allow rapid intraoperative detection of the involved LNs and therefore reduce the rate of MTC persistence. As this method has already been implemented in other surgical/oncological fields and has demonstrated very promising outcomes [22], the low physiological background of [^18^F]DOPA in the head and neck region could enable the detection of small MTC lesions with an intraoperative gamma probe.

The results of the study could also emphasize the impact of Ctn DT on MTC patient survival. Patients with a Ctn DT of less than 1 year were clearly at higher risk of death than patients with a longer Ctn DT. In accordance with these results, Gawlik et al. showed retrospectively that a Ctn DT of less than 2 years is a negative prognostic factor in patients with persistent or recurrent MTC [23].

The present study had some limitations. Although the number of patients included was higher than in previous studies in patients with primary MTC, the small sample size was still the main limitation. Furthermore, the study was limited by its retrospective design. Homogenizing the cohort prospectively and providing an exact LN template in relation to the imaging results will further elaborate the results. In our cohort, specificity and negative prediction of the region-based analysis were affected by these methodological limitations so these parameters cannot yet be transferred into the clinical workflow. Thus, prospective studies with a larger sample are highly recommended to elucidate further the critical diagnostic role of preoperative [^18^F]DOPA examination in patients with MTC.

### Conclusion

[^18^F]DOPA PET/ceCT is a highly sensitive diagnostic method for primary staging of MTC. Preoperative N-staging with DOPA PET/ceCT is significantly more sensitive than ultrasonography for the central and lateral neck levels. Furthermore, DOPA PET/ceCT cN1b status is predictive of postoperative tumour persistence and recurrence. Therefore, radioguided surgical approaches might be an option to improve surgical outcome.
